# Genomic relationship and physiochemical properties among raw materials used for Thai black garlic processing

**DOI:** 10.1002/fsn3.1762

**Published:** 2020-07-09

**Authors:** Piyachat Sunanta, Hsiao‐Hang Chung, Kaewalin Kunasakdakul, Warintorn Ruksiriwanich, Pensak Jantrawut, Surat Hongsibsong, Sarana Rose Sommano

**Affiliations:** ^1^ Interdisciplinary Program in Biotechnology Graduate School Chiang Mai University Chiang Mai Thailand; ^2^ Plant Bioactive Compound Laboratory (BAC) Faculty of Agriculture Chiang Mai University Chiang Mai Thailand; ^3^ Division of Horticulture Department of Plant and Soil Sciences Faculty of Agriculture Chiang Mai University Chiang Mai Thailand; ^4^ Department of Horticulture National Ilan University Yilan City Taiwan; ^5^ Department of Plant Pathology Faculty of Agriculture Chiang Mai University Chiang Mai Thailand; ^6^ Innovative Agriculture Research Center Faculty of Agriculture Chiang Mai University Chiang Mai Thailand; ^7^ Department of Pharmaceutical Sciences Faculty of Pharmacy Chiang Mai University Chiang Mai Thailand; ^8^ Cluster of Research and Development of Pharmaceutical and Natural Products Innovation for Human or Animal Chiang Mai University Chiang Mai Thailand; ^9^ Environment and Health Research Unit Research Institute for Health Science Chiang Mai University Chiang Mai Thailand

**Keywords:** aged garlic, antioxidants, genetic variation, RAPD

## Abstract

Raw materials used for black garlic (BG) processing were collected from the major garlic production areas in Northern Thailand. Five of those were identified as of Thai origin (accession G1–G5), and accession G6 was of the Chinese variety. They were initially analyzed for varietal differences using morphological characteristics and genetic variation. Fresh materials from each accession were dried to the same moisture content (55%–60%) and BG processed at 75°C, 90% relative humidity (RH) for 15 days. Thereafter, physiochemical and chemical profiles were analyzed and compared. The dendrogram from random amplified polymorphic DNA fingerprints grouped G2, G3, G4, and G5 as closely related while G1 and G6 were out‐groups. Prior to BG processing, the pH of fresh garlic was approximately 6.3 and decreased to 3.7, thereafter. The contents of chemical properties were independent with genotypes. BG processing improved phenolic, flavonoid, and antioxidant but the content of thiosulfinate was minimized in all BG samples. Overall, result indicated that garlics grown in Northern Thailand were genotypically variable. BG processing altered physical and chemical appearance, and these changes were independent with the genotypes.

## Graphical abstract

Raw materials used for black garlic (BG) processing were collected. They were initially analyzed for varietal differences using genetic variation, physicochemical, and chemical properties.

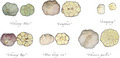

## 1 INTRODUCTION

Garlic is known as culinary herb that had been traditionally used in the recipe of food in many cultures around the world (Bae, Cho, Won, Lee, & Park, 2014; Butt, Sultan, Butt, & Iqbal, 2009; Dufoo‐Hurtado, Huerta‐Ocampo, Barrera‐Pacheco, Barba de la Rosa, & Mercado‐Silva, 2015). For more than thousand years, garlic was recorded in the historical papyrus as medicine to cure many diseases and aliments (Block, 1985). Having been regarded as essential in all kitchens, garlic is today claimed as one of the modern “super foods” with long listed bioactive functionalities (Capasso, 2013; Choi, Cha, & Lee, 2014; Morihara et al., 2007; Nicastro, Ross, & Milner, 2015; Rahman, 2003; Rahman & Lowe, 2006; Sierpina et al., 2013; Zakarova et al., 2014). Approximately 7,000 tons of fresh garlic are produced annually in Thailand and the majority of the production was located mainly in the north including Mae hong son, Lamphun, Lampang, and Chiang rai. In recent years, due to the influx of foreign produce mainly from China, Thai garlic has faced challenges of over‐supply for the domestic consumption. Therefore, attempts have been made toward value‐adding Thai garlic through processing for much preferably healthy products (Bae et al., 2014; Li, Pei, & Qiao, 2014).

Black garlic (BG) processing involves heating the raw material at high temperature and saturated humidity for 14–40 days without additive until the color of the flesh turns to black by either Maillard reaction or caramelization (Bae, Cho, Won, Lee, & Park, 2012; Choi et al., 2014; Hee Kim, Hyun Nam, Rico, & Young Kang, 2012; Lu, Li, Qiao, Qiu, & Liu, 2017). There by, its texture becomes more elastic with sweet–sour taste and less offensive odor (Bae et al., 2014; Choi et al., 2014; Sasaki, 2015; Shin et al., 2008). BG improves amount of reducing sugars, amino acids, and antioxidative compounds which are being recognized as a functional food product in the global market (Kimura et al., 2017; Queiroz, Ishimoto, Bastos, Sampaio, & Torres, 2009). This process also reduces the pungent odor as sulfur volatiles (i.e., allicin) and is converted into water soluble antioxidants which is preferable by consumers who dislike strong garlic smell (Kim, Kang, & Gweon, 2013). The antioxidative compounds in BG in particular possess long lists of functionalities including antibacterial, antibiotic, antifungal, antiviral, anticancer, and antioxidant properties (Choi et al., 2014). Also many studies have shown that aged garlic prevents carcinogenesis, cardiovascular, and age‐related diseases (Rahman, 2003). This process has helped Thai food producers to restore the market position of Thai garlic raw material. Nonetheless, according to our survey, the quality of the BG in Thai markets vastly diverged from one producer to the others. The major problem is that no standardization criteria for raw material quality control have been set up. Previous studies indicated that differences in genotyping had an effect on chemical compositions in both fresh garlic and finished products (Naheed, Cheng, Wu, Wen, & Ding, 2017; Sommano, Saratan, Suksathan, & Pusadee, 2016). Even though other factors could also have forthright effect on BG qualities, for example, temperature, relative humidity during the thermal processing, and pretreatment process (Kimura et al., 2017; Li et al., 2014), the data on varietal differences of raw materials used for BG processing, especially, among the northern Thai food producers are very slim. The research aim of this study was, therefore, to investigate differences in genotyping among the raw materials used for BG processing along with their physiochemical compositions. The key finding from this research will be useful toward raw material selection to ensure the constant quality during commercial processing of Thai BG.

## 2 MATERIALS AND METHODS

### 2.1 Plant material

Unless otherwise stated, all Thai garlic samples listed in Table 1 were collected at the same commercial harvesting stage (between 90–120 days after cultivated) in March 2019 in which leaves appeared dry and the bulb was pinkish‐white, clean without disease and pest damage. They were transported fresh to the laboratory immediately for the experiments. Bulb morphology (viz. size, weight, number of cloves, and their weights) of garlic samples from different cultivation sites in the north of Thailand was recorded (Sommano et al., 2016).

**TABLE 1:** The source of garlic sample

Accession	Common name	Cultivated location
G1	Thai garlic	Sanpatong, Chiang mai (18.615903, 98.910397)
G2	Thai garlic	Li, Lamphun (17.925135, 98.912489)
G3	Thai garlic	Ngaw, Lampang (18.714264, 99.939088)
G4	Thai garlic	Mae‐suai, Chiang rai (19.594376, 99.504031)
G5	Thai garlic	Mae‐sa‐riang, Mae hong son (18.205222, 97.920240)
G6	Chinese garlic	Thai local market

### 2.2 Genetic variation of garlic samples

#### 2.2.1 DNA extraction

Cloves of each sample were separated and propagated as seedling in a wet sponge for 20 days. Young leaf tissue from the seedling was then used for DNA extraction. Total genomic DNA was extracted using the cetyl‐trimethylammoniumbromide (CTAB) method with some modification (Doyle & Doyle, 1986). The extraction buffer [100 mM Tris–HCl pH 8.0, 20 mM ethylene di‐amine tetracetate (EDTA) pH 8.0, 1.4 mM NaCl and 4% (w/v) CTAB] was added to fresh sample ground previously with liquid nitrogen. Thereafter, the mixture was incubated at 65°C for 90 min. Then, the sample was extracted with 600 μl of chloroform:isoamyl alcohol 24:1 (v/v) and centrifuged at 6,800 *g* for 10 min. The supernatant was transferred to a fresh vial, and isopropanol was added to achieve DNA separation. The DNA fraction was air‐dried at room temperature, and then, this pellet was resuspended in 100 μl TE buffer (10 mM Tris–HCl pH 8.0, 1 mM EDTA and pH 8.0). The DNA samples were stored at −20°C prior to random amplified polymorphic DNA (RAPD) analysis.

#### 2.2.2 DNA quantification

DNA was quantified by using L‐vis plate (BMG LABTECH). Moreover, the extracted DNA was run on 1% agarose gel electrophoresis using 1× TBE buffer at 100 V for 30 min by submarine electrophoresis (Mupid^™^‐ONE, Advance) and visualized under LED transilluminator (TT‐BLT‐470; Hercuvan) by staining with Green stain (Cyanagen). The DNA solution was diluted with deionized water for further polymerase chain reaction (PCR) and kept in −20°C until used.

#### 2.2.3 RAPD‐PCR

For RAPD analysis of the genomic DNA, 11 primers were chosen as shown in Table 2. The PCR was adjusted to 25 μl containing 12.5 μl of PCR master mix (HiMedia Laboratories), 1 μl of 25 μM RAPD primer, 1 μl of genomic DNA, and 10.5 μl of deionized water. All the reactions were carried out on a K960 Thermal cycler (Heal Force) using the following program: 1 cycle, 94°C, 4 min; 40 cycles, 94°C, 30 s; 37°C, 30 s; 72°C, 60 s; 1 cycle, 72°C, 10 min (Sommano et al., 2016). Consequently, the sample was separated in a 1.5% agarose gel in 1× TBE buffer. The PCR products were stained with the green stain and run at 100 V for 65 min. The gels were then visualized using the LED transilluminator, accordingly.

**TABLE 2:** Random amplified polymorphic DNA primer sequence used in genetic variation experiment

Primer name	Sequence	Reference
OPA08	GTGACGTAGG	Sommano et al. (2016)
UBC106	AGGAGTCGGA	Sommano et al. (2016)
UBC120	AGACCCTTGG	Sommano et al. (2016)
UBC155	CTGGCGGCTG	Sommano et al. (2016)
UBC184	CAAACGGCAC	Sommano et al. (2016)
UBC215	TCACACGTGC	Sommano et al. (2016)
UBC237	CGACCAGAGC	Sommano et al. (2016)
UBC275	CCGGGCAAGC	Sommano et al. (2016)
OPA14	TCTGTGCTGG	Khar, Asha Devi, and Lawande (2008)
OPR10	CCATTCCCCA	Umaretiya, Hirani, and Marviya (2019)
OPB03	CATCCCCCTG	Parvin, Haque, Al Munsur, and Begum, (2008)
OPB07	GGTGACGCAG	Parvin et al. (2008)

### 2.3 BG processing

Fresh garlic samples were hung in controlled atmospheric condition (5 × 10 m^2^, 29°C and 55% RH) until the moisture content was constant (55%–60%); then, dry leaves and outer tunic were removed. Black garlic was processed in a rice cooker (KS11E; Federal Electric Corporation) using warm mode (at 75°C, 80% RH) for 15 days according to method of Sasaki (2015) with some modification; then, the BGs were removed. The BG samples were lyophilized then ground to powder and stored at −20°C for further phytochemical analyses.

### 2.4 Physicochemical properties

#### 2.4.1 Moisture content

The moisture content of the garlic samples was determined by measuring the weight loss percentage after 12 hr at 105°C in a hot‐air oven according to the Association of Official Analytical Chemists (Cunniff, 1996).

#### 2.4.2 pH and color intensity

Flesh of samples (10 g) was blended with 100 ml of distilled water. Then, the pH was measured using a pH meter (Five easy F20; Mettler Toledo) (Choi et al., 2014), and browning intensity was measured at an absorbance of 420 nm using a spectrophotometer (SPECTROstar Nano; BMG LABTECH; Kang, 2016).

#### 2.4.3 Scanning electron microscopy of cell structure

The garlic cloves were cross‐section into 3 mm‐thick then immediately immersed in cold formalin–acetic acid–alcohol (FAA solution; formaldehyde: acetic acid: ethanol; 1:1:10) for 72 hr. The fixed samples were then dehydrated in a series of aqueous ethanol solutions with successively increasing ethanol concentrations from 30% to 100%, v/v and dried in freeze dryer. Dry samples were coated with platinum using a vacuum sputter coater to increase the conductivity of the samples (Wang & Rhim, 2019). The images were taken using Scanning electron microscope (SEM; TM3000, Hitachi, Ltd).

### 2.5 Chemical properties

#### 2.5.1 Thiosulfinate content

Garlic powder sample (0.5 g) was added to 25 ml of distilled water and shaken for 10 min. The mixture was filtered through Whatman No. 1 filter paper, and the supernatant was separated to a new Erlenmeyer flask. Then, thiosulfinate from the mixture was extracted by using 10 ml of hexane, swirled the mixture gently, and separated the hexane layer. The water layer was re‐extracted with 5 ml of hexane. The first and second extracts were combined, and the absorbance of the hexane solution was measured at 254 nm (Kang, 2016). The thiosulfinate content of the hexane solution was calculated using the following equation:

(1) $$C=\frac{A}{\varepsilon \times b},$$

where *A* is the absorbance; *b* is the path length (cm); *C* is the solution concentration (μM/g); *ε* is the molar absorptivity of thiosulfinate solution at 254 nm (0.014 g/μM cm).

#### 2.5.2 Methanol extraction

The sample powder (0.1 g) was added into 5 ml of 80% methanol in a test tube then heated at 70°C for 30 min. The supernatant was collected after centrifugation at 6,800 *g* for 12 min. The extraction step was repeated five times, and all supernatants were combined. Finally, the resulted extract was adjusted to the total volume of 25 ml with 80% methanol and used as methanol extracts for the subsequent chemical analyses.

#### 2.5.3 Total phenolic content

The total phenolic content was determined using gallic acid as a standard according to the method described by the International Organisation for Standardisation (ISO, 2005) with minor modification. Briefly, methanol extract (30 µl) was mixed with 150 µl of Folin–Ciocalteu reagent then 120 µl of 7.5% w/v NaCO_3_ solution was added, mixed and left in darkness at room temperature for 60 min. The absorbance at 765 nm was measured using the spectrophotometer and total phenolic content was expressed as milligram of gallic acid equivalents per gram of dried garlic sample.

#### 2.5.4 Total flavonoid content

The total flavonoid content was analyzed using catechin as a standard according to the method described by Kim et al. (2013) with some modification. The methanol extract (25 µl) was mixed with 125 µl of distilled water; then, 7.5 µl of 5% NaNO_2_ solution was added. The mixture was allowed to react at room temperature for 5 min before adding 15 µl of 10% AlCl_3_·6H_2_O solution. After 6 min of the incubation, 1 M of NaOH solution (50 µl) and distilled water (27.5 µl) were added. The absorbance was measured at 510 nm using the spectrophotometer. The total flavonoid content was expressed as milligram catechin equivalents per gram of dried garlic sample.

#### 2.5.5 DPPH scavenging radical activity

The free radical‐scavenging activity of BG was determined using the method described by Molan, Flanagan, Wei, and Moughan (2009) with slight modification. The same methanol extract (25 µl) was fused with 250 µl of 0.2 mM DPPH (2,2‐diphenyl‐1‐picrylhydrazyl). After incubated at room temperature in darkness for 30 min, the absorbance was measured at 550 nm using the spectrophotometer. The DPPH scavenging activity was expressed as the DPPH scavenging activity (%) (Choi et al., 2014).

(2) $$\text{DPPH}\;\text{scavenging}\;\text{activity}\;\left(\%\right)=\left[\left(A_{\text{control}}‐A_{\text{sample}}\right)/A_{\text{control}}\right]\times 100,$$

where *A*_control_ is the absorbance of DPPH and *A*_sample_ is the absorbance of sample.

### 2.6 Statistical analysis

All experiment data were expressed as mean (*n* = 9) ± *SE*. One‐way analysis of variance and Tukey's honesty significant difference test were used to determine the significance of the difference among the samples at a confident level of 0.05 using SPSS 24.0 version (SPSS Institute). Principal component analysis (PCA) and cluster analysis of all sample types with physiochemical properties were analyzed using XLSTAT (free‐Trial version, XLSTAT.com).

## 3 RESULTS AND DISCUSSION

### 3.1 Morphology

**FIGURE 1:**
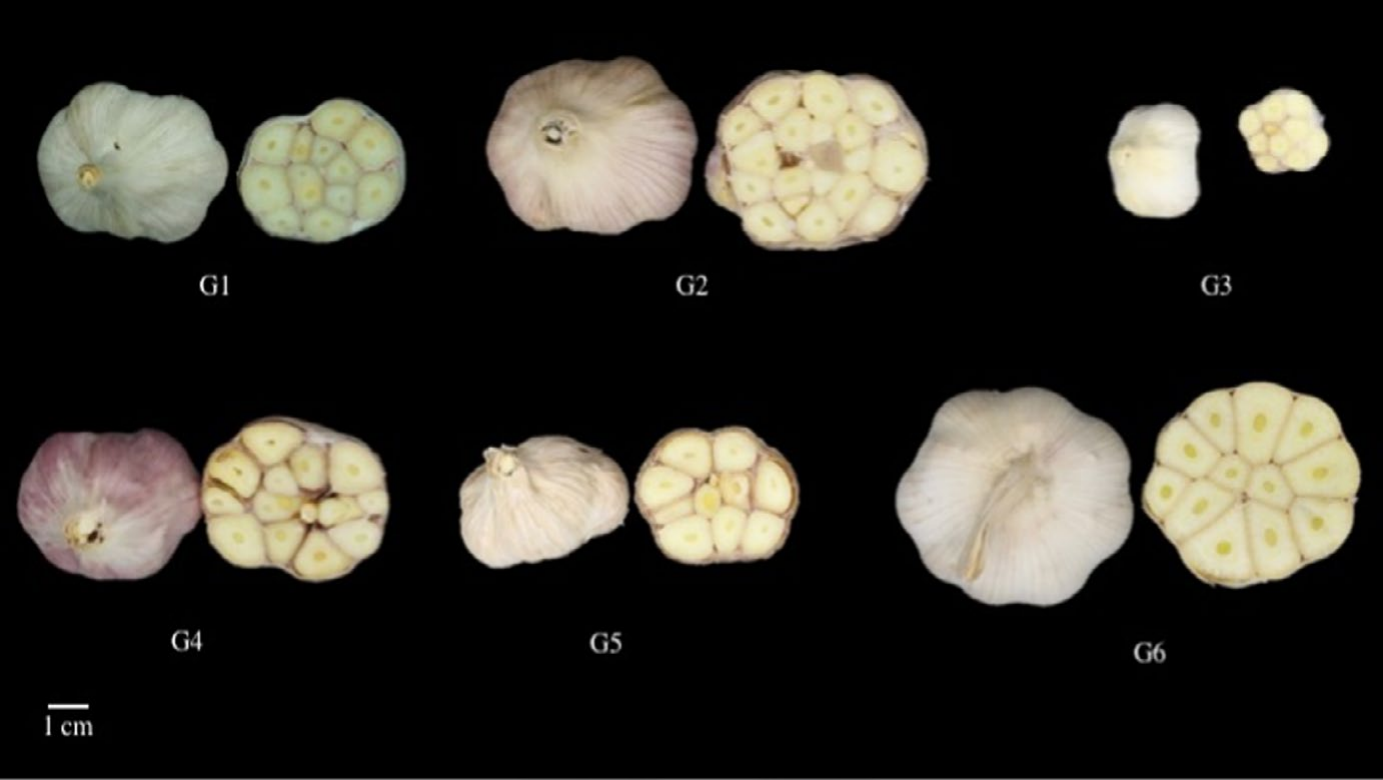
Morphological appearances of garlic bulbs collected from different cultivation sites. G1: garlic collected from Chiang mai, G2: garlic collected from Lamphun, G3 of Lampang, G4 of Chiang rai, G5 of Mae hong son and G6 of Chinese garlic

**FIGURE 2:**
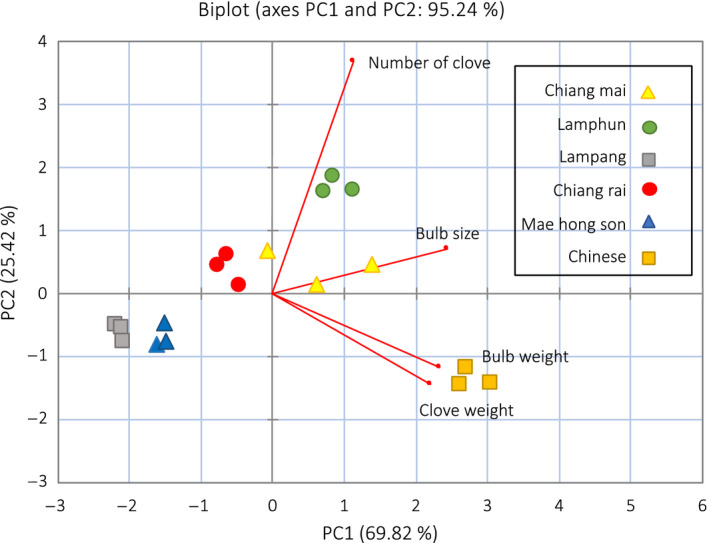
Principal component (PC) analysis biplot analysis of garlic samples and their physiochemical properties

The morphological data of six garlic samples used in this experiment are shown in Table 3. The sizes of bulb and clove of foreign sample (G6) was the biggest as compared with the others. All samples were globose bulbs with circumference 8–15 cm consisting of 10–20 individual cloves. G1, G2, and G4 contained more than 15 cloves while G3, G5, and G6 owned approximately 10 cloves. The weights of individual clove among the Thai cultivated samples were not significantly different (~0.6–0.9 g). The tunic color illustrated the differences within the samples as shown in Figure 1. While G1, G3, G5, and the foreign sample had white tunic, the garlic cultivated in Lamphun (G2) was of pink‐white in color. Garlic grown in Chiang rai province had, however, white‐purple tunic. The physiochemical properties of raw garlics are illustrated in Table 4. Harvested right‐off the field, the moisture content of all samples was usually different depending on their maturity stages. It appeared that fresh garlics of Lamphun and Lampang had the highest moisture content (>58%). Relationship between samples and fresh garlic physicochemial properties was evaluated using PCA biplot analysis as shown in Figure 2. The PCA space distributes 69.82% in PC1 and 25.42% in PC2. The data were divided into three clusters across the PCA space, the first cluster included samples of Lampang and Mae hong son. The second cluster contained samples of Chiang Mai, Lamphun, and Chiang Rai which were higher in number of clove and bulb size while Chinese garlic was separated into different cluster due to bulb and clove weights. Chen et al. (2013), however, urged that morphological traits alone were unable to fully describe garlic variety, and thus, differences in genotyping should be analyzed.

**TABLE 3:** Morphological appearances of garlic samples

Sample	Cultivated area	Bulb appearance	Bulb size (cm)	Bulb weight (g)	Clove number	Clove weight (g)	Cultivated duration
G1	Sampatong, Chiang mai	Oval	12.52 ± 0.21^b^	13.9 ± 0.83^c^	18 ± 1^c^	0.94 ± 0.30^a^	November–March
G2	Li, Lamphun	Oval	13.00 ± 0.49^b^	15.49 ± 1.65^c^	19 ± 2^c^	0.91 ± 0.10^a^	November–March
G3	Ngao, Lampang	Oval	8.60 ± 0.08^a^	4.53 ± 0.15^a^	9 ± 1^a^	0.59 ± 0.05^a^	November–March
G4	Mae‐suai, Chiang rai	Oval	11.52 ± 0.57^b^	10.72 ± 1.23^bc^	15 ± 1^bc^	0.88 ± 0.06^a^	November–March
G5	Mae‐sareang, Maehongsorn	Oval	9.80 ± 0.27^a^	6.15 ± 0.80^ab^	9 ± 1^a^	0.83 ± 0.05^a^	November–March
G6	China	Round	15.06 ± 0.27^c^	33.80 ± 1.67^d^	11 ± 0^ab^	2.23 ± 0.24^b^	No data

Note

Values are mean ± *SD*. Values followed by different letter in the same column are significantly different (*p* < .05) by Tukey's honesty significant difference test.

**TABLE 4:** Physiochemical and chemical properties of fresh garlic and black garlic

Samples	G1		G2		G3		G4		G5		G6	
	Fresh	Black	Fresh	Black	Fresh	Black	Fresh	Black	Fresh	Black	Fresh	Black
Physiochemical properties												
Moisture content	57.54 ± 0.45^b^	32.41 ± 2.87^c^	60.48 ± 0.89^c^	25.96 ± 1.40^ab^	58.62 ± 0.43^c^	24.56 ± 1.91^a^	55.86 ± 0.32^a^	25.80 ± 0.85^a^	55.52 ± 0.36^a^	30.63 ± 0.62^b^	56.36 ± 0.16^ab^	53.12 ± 2.32^c^
pH	6.49 ± 0.02^e^	3.79 ± 0.03^c^	6.38 ± 0.01^c^	3.63 ± 0.01^b^	6.27 ± 0.00^a^	3.80 ± 0.02^c^	6.39 ± 0.01^b^	3.53 ± 0.02^a^	6.55 ± 0.03^c^	4.04 ± 0.02^d^	6.32 ± 0.02^ab^	3.68 ± 0.01^b^
OD420	0.21 ± 0.00^a^	5.22 ± 0.22^bc^	0.23 ± 0.02^a^	6.55 ± 0.48^bc^	0.23 ± 0.06^a^	8.25 ± 0.96^c^	0.24 ± 0.00^a^	6.05 ± 0.42^bc^	0.25 ± 0.00^a^	6.52 ± 0.03^ab^	0.33 ± 0.01^b^	3.64 ± 0.28^a^
Chemical properties												
Thiosulfinate	12.62 ± 0.71^c^	3.19 ± 0.20^a^	9.53 ± 0.62^b^	2.74 ± 1.06^a^	16.70 ± 0.57^d^	3.10 ± 0.70^a^	11.62 ± 0.01^c^	2.40 ± 0.24^a^	9.44 ± 0.05^b^	3.650.24^a^	9.23 ± 0.06^b^	2.860.69^a^
Total phenolic	0.59 ± 0.05^a^	5.32 ± 0.58^c^	0.60 ± 0.02^a^	4.58 ± 0.22^b^	0.46 ± 0.04^a^	6.32 ± 0.23^d^	0.53 ± 0.04^a^	6.26 ± 0.12^d^	0.85 ± 0.01^a^	6.69 ± 0.15^d^	0.22 ± 0.02^a^	5.58 ± 0.40^c^
Total flavonoid	0.30 ± 0.09^a^	0.79 ± 0.09^c^	0.27 ± 0.02^a^	1.04 ± 0.07^d^	0.35 ± 0.09^a^	1.12 ± 0.13^de^	0.28 ± 0.06^a^	1.30 ± 0.08^e^	0.14 ± 0.01^a^	0.57 ± 0.04^b^	0.34 ± 0.03^a^	0.97 ± 0.10^cd^
DPPH scavenging activity	16.07 ± 0.10^ab^	91.52 ± 3.69^de^	15.83 ± 0.07^ab^	87.12 ± 2.09^cd^	9.28 ± 0.19^a^	95.49 ± 1.21^e^	16.22 ± 1.73^ab^	94.59 ± 2.20^de^	19.95 ± 4.21^b^	90.62 ± 0.27^cd^	12.17 ± 0.06^a^	83.12 ± 4.93^c^

Note

Values are mean ± *SD*. Values followed by different letter in the same row are significantly different (*p* < .05) by Tukey's honesty significant difference test.

Abbreviation: DPPH, 2,2‐diphenyl‐1‐picrylhydrazyl.

### 3.2 Genetic variation of garlic samples

**FIGURE 3:**
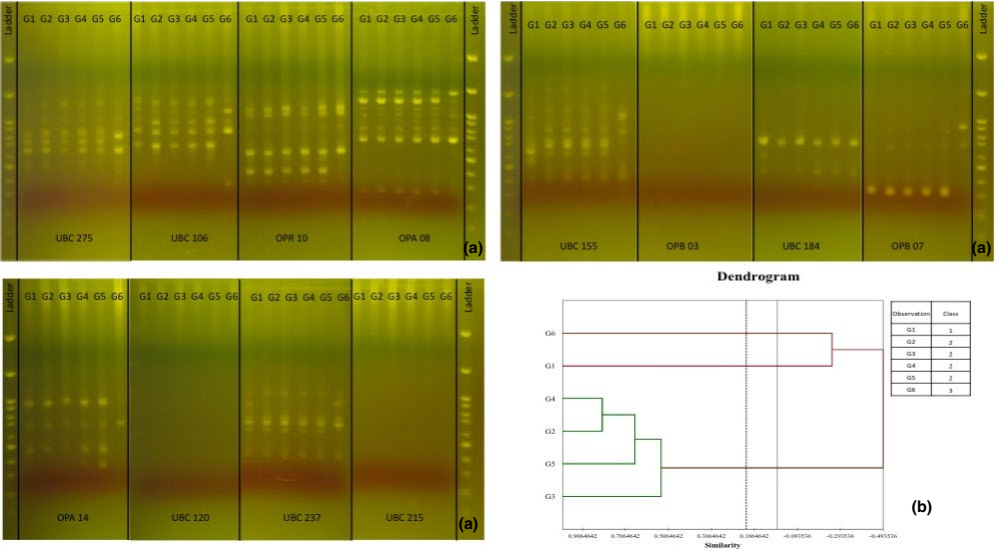
(a) The random amplified polymorphic DNA (RAPD)‐PCR fingerprints of all garlic samples using 12 randomly primers (b) The dendrogram of garlic samples generated from similarity matrix of RAPD data

The RAPD‐PCR fingerprints were generated from DNA extract of all garlic samples using 12 randomly 10‐oligonucleotide primers as shown in Table 2. Only nine primers, however, provided band patterns. Results indicated that there were 69 fragments based on the RAPD (Figure 3a) which later on were used to create a dendrogram as shown in Figure 3b. The dendrogram divided garlic samples into three groups: the first group includes G1 from Chiang mai. The second cluster contained G2, G3, G4, and G5 (similarity 66%–82%) as for the garlic samples cultivated in Lamphun, Lampang, Chiang rai, and Mae hong son, provinces, respectively. The G6 of the foreign type was separated into different cluster. The garlic samples cultivated in Chiang Mai and China were 49% dissimilarity compared to the first group. Considering their morphological differences, the genotyping result does not relate with the morphological data. This advised that the feature cannot be accounted for genotypic alteration. These results were in associated with the study of Buso et al. (2008) who found that the genetic variations of garlic grown in Brazil were not related to bulb shape, bulb color, and number of clove. In addition, the study of Shree, Singh, and Nitish (2014) showed that the growing environment had significant affected the characters of garlic viz., weight of bulb, number of cloves per bulb, diameter of clove, and average weight of clove. Ebrahimi, Mohammadi‐Nejad, Baghizadeh, and Abdolinejad (2011) explained that the genetic variation could be as a result of mutation in plants to adapt to the growing environments. Chen et al. (2014) also found that garlics from the same geographic origin were not grouped together in the same genetic cluster. This finding is, however, in contrast with work done by Sommano et al. (2016) who confirmed close relation between Chinese varieties with Thai garlic.

### 3.3 Physicochemical properties of BG

**FIGURE 4:**
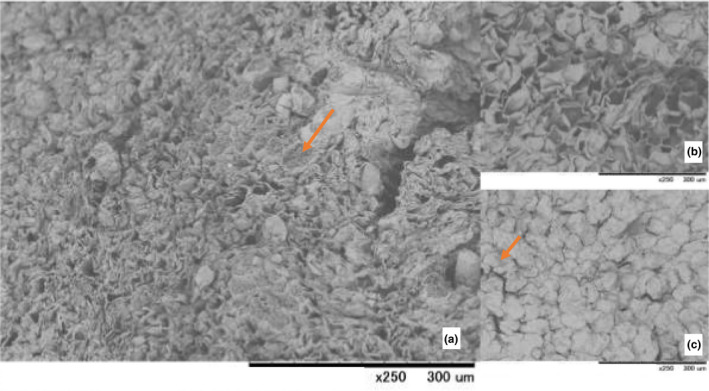
(a) Scanning electron microscope images of black garlic from Lampang origin (b) fresh raw material of Lampang origin (G3) and (c) black garlic from raw material of the Chinese origin. Arrows indicate blackening pigments accumulated within cell structure after processed

After BG processing, the moisture content of BG, however, was independent with the initial moisture contents but variety differences had greater impact on the finished product moisture content, average 30% in Thai garlic samples and 53% in foreign garlic (Table 4). This was in‐line with the work of Naheed et al. (2017) who advised that the varietal variation could affect weight loss during processing. Geometrical shape of the bulb also influences moisture loss activity which much more labile in the outermost layers reached there after the most difficult part of the inner core (Bloem, Haneklaus, & Schnug, 2011). From our result, the Chinese cultivar gave the lowest water loss during the processing as the bulb was 1.3–1.8 times bigger than other Thai cultivars. SEM images illustrated that the greater moisture content of the product could lead to higher degree of damaged to the cell structure (Figure 4).

The pH of all fresh garlics was detected just below seven and decreased thereafter BG processing (~3.8) as shown in Table 4. The pH was also varietal independent as all sample types possessed different pH values before and after processed. To this, Choi et al. (2014) explained that depending on the chemical compositions, heat treatment encouraged the breakdown of the products to much more acidic ingredients of carboxylic acid and phenolic acid through Maillard reaction, caramelization, and to the less extend with polyphenol oxidation (Bae et al., 2014). Maillard reaction is a nonenzymatic reaction between reducing sugars and amino group which was naturally produced during heat processing (Yilmaz & Toledo, 2005). This generated the dark brown pigments as a result of the reaction (Choi et al., 2014). In BG, fructan is mainly responsible for this reaction. Rate of the reaction depends on many factors, for example, temperature, time, pH, and water activity. Cuzzoni, Stoppini, and Gazzani GandMazza (1988) studied the effect of water activity on heated ribose‐lysine and glucose‐lysine, and the result showed that at high water activity, the rate of Maillard reaction was low. Bae et al. (2014) suggested that decrease in pH is in association with browning mechanism during heat process. The UV absorbance at 420 nm is regularly used to measure the brown color formation of biochemical reaction (Yuan, Sun, Chen, & Wang, 2018). In the experiment, brown color intensity increased the greatest in G3 but less in other Thai samples. The browning intensity was the lowest in the foreign garlic (G6), which apparently was well corresponded with the higher moisture content of the BG (53%). Browning intensity could therefore be as consequences of low pH and higher moisture content. Accumulation brown pigments was formed as a result of Maillard reaction between the products of cell structure as shown in Figure 4a,c (red arrows pointed).

**FIGURE 5:**
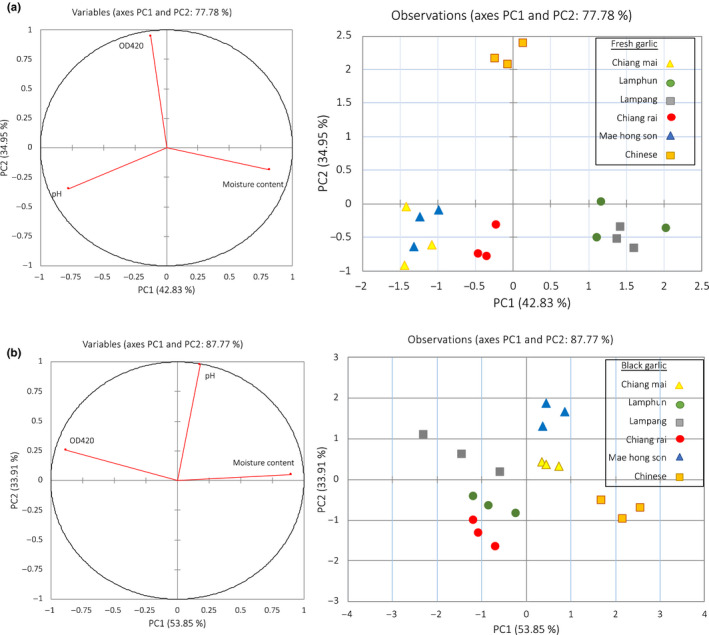
Principal component (PC) analysis observation plot of garlic samples based on their physiochemical properties (a) fresh garlic (b) black garlic

From the PCA analysis which deposits 42.83% in PC1 and 34.95% in PC2, the data were categorized into three clusters (Figure 5a): the first cluster included samples of Chiang Mai, Chiang Rai, and Mae hong son, the second cluster consisted of samples cultivated in Lamphun and Lampang while Chinese garlic was separated. Biplot factor analysis illustrated that the first cluster was distinct from the others by pH. Samples of cluster 2 were separated by initial moisture content, and Chinese garlic was elucidated to have different browning intensity as determine by OD420. The PCA biplot factor analysis of BG samples and physicochemical properties were demonstrated in Figure 5b. The PCA accounts for total of 87.77% in PC1 and PC2. All samples illustrated their own physical characteristics except those of Lamphun, Lampang, and Chiang Rai that were not clearly separated. These confirmed that genotype had no impact on physiochemical properties after BG processing.

### 3.4 Chemical properties

Total phenolic, flavonoid, and sulfur compounds are the main antioxidative compounds in garlics (Leelarungrayub, Rattanapanone, Chanarat, & GebickiJM, 2006). BG processing marked significant change in chemical properties in all garlic samples (Table 4). Thiosulfinate presents the unique garlic flavor, and the content usually decreases during heat processing due to the alteration of thiosulfinate to S‐allyl cysteine, S‐allyl mercapto‐cysteine, and arginine. Kang (2016), Choi et al., (2014) and Zhang, Lei, Zhu, and Zhang (2013) reported the beneficial antioxidant, anticancer, antihepatopathic, and neurotrophic activities of BG. Our result reported that the level thiosulfinate was altered in all fresh garlic of different cultivars; however, after heat treatment the levels in all samples declined up to 80%. Among different varieties of BG, thiosulfinate was not significantly different.

Total phenolic content in the BGs was significantly higher than that of fresh garlics and the content increased up to 13‐fold after BG processing as shown in Table 4. BG3, BG4 and BG5 showed the highest total phenolic content approximately 6.42 mg/g of all processed samples. Kang (2016) and Robards, Prenzler, Tucker, Swatsitang, and Glover (1999) explained that total phenolic content increased due to the release of the bound of polyphenol compound to phenolic acid after high temperature processing. Cheng, Dai, Zhou, Yang, and Liu (2007) also reported that the phenolic compounds of bound form can be separated from cell wall by heat processing. As a result, the content of oxidative phenolic acid is usually higher with this process. This result was compatible with the study of Scalzo, Iannoccari, Summa, Morelli, and Rapisarda (2004), who reported that high temperature processing increased phenolic substances of fruit and vegetable crops.

Flavonoids belong to the phenolic group, and the flavones are generally found to a greater extent in fresh garlic. The total flavonoid content was similar among the fresh garlic samples (~0.28 mg/g). BG processing increased the content of the total flavonoid and the level was much higher in the BG4 (~1.3 mg/g). Flavonoid was higher in BG wherewith the heat treatment discharged the bound polyphenolic and flavonoid compounds (Choi et al., 2014). Flavonoid content alteration depends on the processing condition and their sensitivity to heat treatment (Ioannou, Hafsa, Hamdi, Charbonnel, & Ghoul, 2012). During the BG processing, the flavanols and flavanones are the main metabolites as they illustrate thermostable characteristic (Kim et al., 2013).

The DPPH radical‐scavenging activities increased up to 86.21% after heat processing. The study of Stratil, Klejdus, and Kubáň (2006) demonstrated that there was a greatly significant relationship between total phenolic content and antioxidant activity. Accordingly, the trend of DPPH scavenging activity in this study was assimilative to total phenolic and flavonoid content. Moreover, all those properties were correlated with processing temperature (Bae et al., 2014). Many studies demonstrated that antioxidants played an important role in preventing diseases caused by free radicals as well as the memory and nervous systems.

**FIGURE 6:**
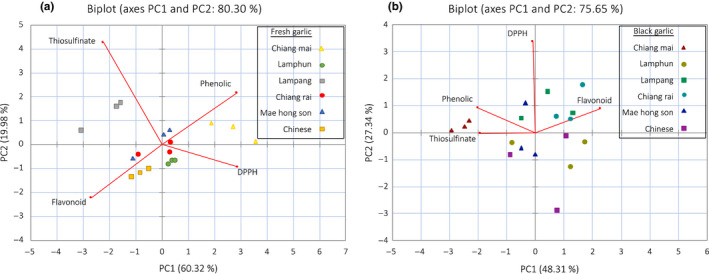
The principal component (PC) analysis biplot analysis of garlic samples based on their chemical properties. (a) Fresh garlic (b) black garlic

We used PCA to explain relationship of the samples based on their chemical properties. For fresh garlic samples, the PCA warranted total of 80.30% PC1 and PC2 as shown in Figure 6a. From the biplot analysis, it only showed the distinct separation of Lampang and Chiang Mai, while other samples were grouped together. However, after aged processing we were only able to see the separation of Mae hong son garlic in Figure 6b. Again, the results established that genotype had no influence on chemical property changes after BG processing.

## 4 CONCLUSION

In an attempt to explore the genotype differences of raw materials used in BG processing in association with the phytochemical properties before and after processing, this study demonstrated that there were morphological differences and genetic variations between garlic samples cultivated in different areas of the northern Thailand. In addition, the physicochemical and chemical properties of both fresh and BGs were dependant with genotype. Future direction should look into the other factors that may influence BG qualities such as postharvest handling condition of raw material prior to processing.

## 5 ETHICAL GUIDELINES

Ethics approval was not required for this research.

## CONFLICT OF INTEREST

The authors declare no conflict of interest with respect to the research.

## Data Availability

The data that support the findings of this study are available from the corresponding author upon reasonable request.
